# Cutting Edge: Biomarkers for Chronic Spontaneous Urticaria

**DOI:** 10.1155/2018/5615109

**Published:** 2018-11-21

**Authors:** Marco Folci, Enrico Heffler, Giorgio W. Canonica, Raffaello Furlan, Enrico Brunetta

**Affiliations:** ^1^Department of Biomedical Sciences, Humanitas University, Milan, Italy; ^2^Internal Medicine, Humanitas Research Hospital, Milan, Italy; ^3^Personalized Medicine, Allergy and Asthma, Humanitas Research Hospital, Milan, Italy

## Abstract

Chronic spontaneous urticaria (CSU) is defined by the appearance of wheals and a variable presence of angioedema which persists for at least 6 weeks. It represents the most common subtype of chronic urticaria and is gaining importance in civil society because of its association with impaired quality of life. Moreover, CSU has a growing impact on national health systems representing a great burden due to its variable rate of response to the approved therapies. In this scenario, the identification of clinical and molecular biomarkers is of pivotal importance. Some groups are trying to detect molecules which would be able to help clinicians in reaching a proper diagnosis; additionally, the opportunity to describe disease severity which leads to cluster patients in different groups could fill the gap in the numerous unmet clinical needs. Several biomarkers are currently being studied with the purpose to predict the response to a defined therapy; unfortunately, none of them are ready to be translated from bench to bedside.

## 1. Introduction

Urticaria is a skin disease characterized by the development of wheals, angioedema, or both. It can have an acute course or become chronic, lasting longer than 6 weeks [1], and in many cases persists several months or years [2]. Another clinically relevant classification is based on whether the wheals are induced by a trigger which defines urticaria as inducible or not and hence referred to as “chronic spontaneous urticaria” (CSU) [1]; of note, up to 45% of patients with CSU, apart from spontaneous wheals, may also develop typical clinical manifestations induced by a specific trigger [3].

It has been estimated that one in every 100–200 European adults has been diagnosed with chronic urticaria, with CSU and females accounting for about two-thirds of the cases [4, 5].

As a highly prevalent disease, CSU has a great impact on the general population representing a burden for national health systems due to its variability and yet unknown pathogenesis. In fact, more and more patients undergo several clinical examinations and medical tests before a diagnosis is reached and the correct therapy is administered. Moreover, CSU significantly impairs the quality of life and in some cases is only partially responsive to the newest therapies. Concerning the long duration of CSU on average, prognostic factors have been widely investigated, but only few have been found to be significantly associated with longer disease duration: its severity [6, 7], the concurrent presence of angioedema [7, 8] or inducible urticaria [8, 9], the finding of positive response against their own serum in the so-called “autologous serum skin test” (ASST) [9, 10], and concomitant arterial hypertension [11].

The treatment of CSU aims to achieve complete symptom relief while using as little medications as possible [1]: in fact, the International Guidelines suggest a progressive increase in treatment level in cases of incomplete response, starting with a single standard daily dose of second-generation H1-antihistamines that can be increased up to 4 daily doses before considering alternative add-on therapeutic strategies [1]. In patients not responding or partially responding to four doses of antihistamines, the use of omalizumab, a humanized anti-IgE antibody, is suggested as the first add-on treatment alternative [1], as its effectiveness has been proved by both large registration trials [12] and real-life studies [13–17]. For those few patients who do not respond to omalizumab, immunosuppressants (mainly oral cyclosporine) or a plethora of other unlicensed therapeutic strategies may be considered [1].

**Figure 1:**
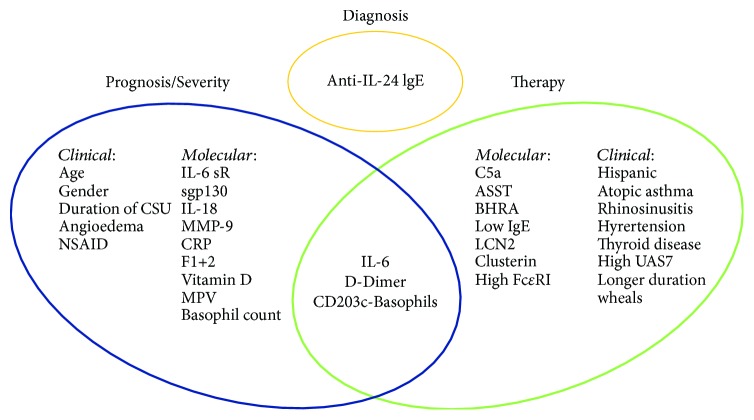
Clinical and molecular biomarkers in CSU.

From this brief introduction, it is clear that CSU is a heterogeneous disease with several different possible clinical characteristics, associated factors, and different degrees of response to the given drugs. Therefore, it could be informative and clinically important to identify the biomarkers able to classify patients according to their phenotype, possibly identifying underlying immunological mechanisms (and therefore setting the disease “endotype”), to stratify patients according to their severity and prognosis, and to identify best responders to any given therapy, particularly to biologics such as omalizumab (Figure 1).

The aim of this review article is to summarize the evidence on clinically relevant biomarkers in CSU.

## 2. Diagnosis and Phenotypic and Endotypic Characterization

From a clinical point of view, CSU is quite heterogeneous. Patients may or may not have recurrent angioedema associated with the chronic presence of wheals, or any trigger of inducible urticaria associated with spontaneous appearance. The disease may differ by its severity, by its impact on the patient's quality of life, and by the clinical response to any given therapy [5]. Moreover, several underlying mechanisms sustaining CSU have been suggested: from an autoimmune hypothesis (IgG antibodies against the Fc*ε*RI alpha subunit of the high-affinity IgE receptor have been found in more than one-third of CSU patients [18], while 5–10% exhibit the production of IgG against IgE antibodies [19]; moreover, up to 33% of CSU patients have anti-thyroid autoantibodies [20]) to an activation of the extrinsic coagulation pathway [21, 22] or even a possible class E immunoglobulin- (IgE-) mediated autoimmunity [23]. From a diagnostic point of view, CSU is a diagnosis of exclusion, and currently, there are no biomarkers specific for the disease. Few but promising data come from a recent study in which Schmetzer et al. discovered a strong association between the presence of anti-interleukin 24 (IL-24) IgE and CSU [24]. In fact, the study describes that in addition to IL-24 being common, it is also a specific autoantigen of IgE which are significantly represented in the serum of CSU patients compared to healthy controls [24] (Table 1).

Considering this extreme heterogeneity, it should be easy to classify patients in different clinical subgroups (phenotypes) or based on the underlying mechanism (endotypes). However, despite the many studies which report differences in biomarkers between subgroups of CSU patients, only few variables seem strong enough to be used as possible phenotypic/endotypic biomarkers. Patients with positive ASST, for instance, are frequently characterized by reduced basophil count and high levels of IgG anti-Fc*ε*RI, while weaker evidence associates ASST-positive patients with higher blood levels of IL-17 and the mean platelet volume (MPV) [25]. The real clinical meaning of distinguishing patients by these variables is still unknown and therefore clinically irrelevant.

Other phenotypic classifications, based on other biomarkers or clinical characteristics and based on larger registrative studies, are inconsistent and irrelevant from the clinical and therapeutic point of view. Therefore, it has not been possible so far to define precise phenotypes or endotypes of CSU, and further studies are needed to understand if they really exist and whether any eventual subgroup classification could improve the management of CSU patients.

## 3. Severity and Prognosis

The lack of clinical and laboratory biomarkers able to define the severity and prognosis usually leads to frustration for patients as well as for physicians. For those reasons, it seems crucial to analyze a big dataset in order to detect possible features which can, used alone or in a multiparametric score, predict the history of the disease.

### 3.1. Clinical Biomarkers

Different studies have described different clinical markers of severity and prognosis; few datasets show how age could be inversely associated with disease severity; the less old the patient is, the less severe the disease [2]. Being female seems to be a predictor of longer time to remission [26] and quality of life impairment [27]; in fact, as demonstrated by Amsler et al., urticaria onset occurs more often after puberty, is worsened by pregnancy, and could be reactivated by hormonal contraception [28]. However, the authors do not describe sex hormones as a pathogenic factor but only as a strong trigger in a small subset of patients [28]. The duration of urticaria was defined as another factor related to severity [26]; indeed, a greater improvement is seen, after the prescription of an efficacious therapy, in disease with a duration less than a year compared to a longer period [2]. The presence of angioedema was analyzed by several groups and was identified as a factor strictly related to a less favorable prognosis [7, 29]. There is little evidence regarding a link between a positive ASST and a severe disease. In contrast, exacerbations occurring with the use of aspirin or nonsteroidal anti-inflammatory drugs (NSAID) have been widely described as being related to a more severe and chronic disease [30, 31] (Table 1). An obvious clinical marker of severity is the lack of response to treatment. Increasing evidence demonstrates how the failure to respond to high-dose antihistamines, up to four times the usual dose as indicated by guidelines [1], is related to severe CSU [32]. Those patients usually require omalizumab or other immunosuppressive agents as a second-line treatment. Unfortunately, there are no markers that may help predict a poor response to first-line therapy.

### 3.2. Molecular Biomarkers

The purpose of studying blood molecules derives from the clinical need to measure the severity of the disease and foresee its evolution. Several putative biomarkers have been investigated with different results in the last decade; however, up to date, none of them filled the gap, leaving the clinician alone during the assessment of the patients (Table 1). The lack of strength and specificity in the majority of the studies can be attributed not only to the retrospective approach but also to the low number of patients considered and the interference of confounding factors. International effort is required for planning large multicenter studies which should avoid those biases and increase the potency.

#### 3.2.1. C-Reactive Protein

Nowadays, one of the most important biomarkers is C-reactive protein (CRP). Several studies found its levels to be elevated in CSU patients in comparison to healthy controls [33]. Kolkhir et al. demonstrated retrospectively on a large cohort of CSU patients how high values are associated with urticarial activity, life impairment, autologous serum skin test, and arterial hypertension [34]. Moreover, some evidence in scientific literature underlines the correlation between acute but also new onset urticaria and CRP levels, suggesting the use of this blood test as a marker of short-disease duration [35, 36]. The link between CRP and CSU and a higher risk of arterial hypertension was demonstrated considering only older patients probably due to a higher prevalence of hypertension in this phase of life [37–40].

#### 3.2.2. Interleukin 6

With regard to the hyperactivation of proinflammatory pathways in CSU, another molecule that was investigated is interleukin 6 (IL-6). It is well known how IL-6 plays a pivotal role in immune and inflammatory responses through its soluble receptor (IL-6 sR) and signal-transducing membrane glycoprotein 130 (gp130) [41–43] which is counteracted in the bloodstream by soluble gp130 (sgp130) [44]. Kasperska-Zajac et al. demonstrated a significant correlation between IL-6 and CRP; furthermore, this group found higher levels of IL-6 sR in CSU patients in comparison to healthy controls [45]. This finding suggests an overactivation of the IL-6 transduction pathway which may enhance the disease activity promoting chronic inflammation. Additionally, the authors demonstrated a significant increase in sgp130 in CSU patients and how this IL-6-negative regulator is strictly related to CSU severity even if its biological significance is not yet known [45].

#### 3.2.3. Vitamin D

Vitamin D has a well-known mechanism of action in mineral homeostasis and bone metabolism; however, it has been demonstrated to have potential clinical implications in determining susceptibility to autoimmune disease because of its immunomodulatory activity [46]. Variation in vitamin D blood levels was investigated as a putative predisposing factor in several autoimmune and allergic diseases such as atopic dermatitis and asthma [47]. For those reasons, this molecule was considered as a potential biomarker of disease severity in CSU by Woo et al. [48]. The study, published in 2015, analyzed serum levels of 25-(OH) vitamin D from 72 patients with CSU, 26 with acute urticaria, and 26 with atopic dermatitis matched with 72 healthy controls [48]. Vitamin D was found to be low in all groups in comparison to controls, even if CSU patients showed the lowest mean levels and the highest proportion of critically low titers. The group underlined also an inverse association between CSU activity score and disease duration [48].

#### 3.2.4. D-Dimer and F1+2

Plasmatic markers of thrombin generation and fibrinolysis were found to be abnormally high in CSU patients as demonstrated by Asero et al. [21, 22]. In those studies, a significant proportion of patients, out of the total examined, showed elevated levels of the fragment F1+2 [49] as well as D-dimer plasma levels, proving that there is an activation of fibrinolysis [21, 22]. Moreover, patients showing elevated D-dimer and/or F1+2 plasma levels showed a more severe disease in most cases [21, 22]. Albeit the relatively small number of patients, 21 in the first study and 37 in the second, the association with D-dimer and F1+2 was statistically strong. Moreover, the results were replicated by Takeda et al. [50] suggesting the need to evaluate these molecules on large series of patients in order to validate them as biomarkers.

#### 3.2.5. Medium Platelet Volume

MPV was related to chronic urticaria by Confino-Cohen et al. [36] in a study on a large cohort of patients. They selected almost 13,000 patients who were diagnosed by allergy or dermatology specialists over 17 years in Israel. For each patient, they collected epidemiological data and medical history as well as inflammatory-related serologic markers. The study group was compared to a control group of 10,000 healthy patients. The study showed a statistically positive association between high MPV in patients affected by chronic urticaria compared to healthy controls [36]. Another group found a similar association on a smaller dataset [51]. Indeed, Magen et al. demonstrated a strong correlation between high MPV in CSU patients and positive ASST results [51]. Additionally, this finding was related to clinical severity which suggests the use of MPV as a potential marker of disease activity underlining the role of platelets as indirect expression of systemic inflammation. Similar studies were published correlating higher MPV values with active inflammatory disease, such as small studies conducted on patients with rheumatoid arthritis [52, 53] or on individuals with newly diagnosed celiac disease [54]. Despite the evidence validated on a large cohort of patients, prospective studies focusing on disease severity which measure MPV as a potential biomarker are still lacking.

#### 3.2.6. Basophil Count and Activity

Other findings come from analysis performed on relatively small groups of CSU patients which underline how peripheral basophil count and its activity could be related to the disease clinical features. A reduction in the total number of those leukocytes on the peripheral blood was linked with disease activity as shown by Grattan [55]. This group demonstrated that there was a negative linear correlation between basophil numbers and UAS in untreated chronic urticaria patients [55], probably explained by the recruitment of those cells to skin lesion sites [56]. Indeed, the use of systemic steroids which usually reduces skin wheals is related to an increase of blood basophil number probably due to an inhibition of basophil recruitment [57–59]. Moreover, Ye et al., analyzing basophil surface receptors, have demonstrated on a group of 82 patients the relationship between the percentage of CD203c-expressing basophils and clinical parameters of severity [60]. In this study, basophils which express CD203c were significantly increased in the blood of patients with severe urticaria compared to those with nonsevere disease and normal controls [60]. As is well known, the presence of the CD203c on basophil membrane is a standardized proof of cell activation; in this fashion, it could be applied to several clinical settings as a marker of severe CSU, guiding not only the therapeutic strategy but also the intensity of the approaches used. Evidence of basophil involvement in CSU pathogenesis comes from studies on the capability to release histamine through the activation of the Fc*ε*RI pathway [61–64]. An impaired quality of life measured by a higher severity of itch, higher frequency of emergency department visits, and longer disease duration is related to the basophil responder phenotype to anti-IgE [65]. However, if patients were properly treated reaching clinical remission, basophils would shift toward normalization of basophil IgE receptor-mediated histamine release with correction of peripheral blood number [62, 66].

#### 3.2.7. Interleukin 18 and Metalloproteinase-9

Limited data underline a direct correlation between interleukin 18 (IL-18) and CSU severity [67–69]. IL-18 is a member of the IL-1 family, a set of proinflammatory cytokines, which was initially identified as a major inducer of interferon-*γ* in natural killer cells and T helper 1 lymphocytes [70]. IL-18 activity is negatively regulated by a soluble molecule called IL-18BP which binds the cytokine and prevents IL-18 interaction with cell surface receptors [71]. IL-18 could be involved in CSU pathogenesis recruiting and activating eosinophils in inflamed tissue generating a loop which stimulates the secretion of IL-8; nonetheless, further studies are needed to confirm those findings [72]. Metalloproteinase-9 (MMP-9) is an enzyme responsible for tissue remodeling due to its capacity to cleave collagen, which is the main component of the basement membrane. MMP-9 can be synthesized by many types of cells, including macrophages, neutrophils, T cells, and mast cells, and has an important role modulating inflammatory processes by its activity; in fact, MMP-9 can cleave proinflammatory chemokines and cytokines influencing migration and activation of immune cells [73–76]. Some evidence demonstrates high levels of MMP-9 in CSU patients [77–79]; however, because of conflicting results, more studies are needed to prove those data.

## 4. Treatment Efficacy

Nowadays, there are different treatment lines to achieve CSU remission as recommended by International Guidelines [1]; however, predicting the efficacy of a therapeutical scheme before prescribing the drug seems pivotal to reach remission, improve the health of the patients, and avoid waste. Several studies tried to identify and cluster groups of patients by clinical efficacy to a defined drug; nonetheless, foreseeing the response remains an unmet need in CSU treatment (Table 2).

### 4.1. Antihistamine Therapy (AH)

Several prognostic factors for antihistamine-resistant CSU (AHr-CSU) have been studied over the last few years; however, their clinical role seems currently limited primarily due to the definition of CSU itself as well as in comparison with biomarkers for omalizumab-resistant CSU (Table 2). In a retrospective analysis on the clinical and demographic information of patients with CSU, Sánchez Borges et al. reported that AHr-CSU was more frequent among Hispanic female patients (aged 20 to 59 years) and usually associated with other clinical indicators of severity such as atopic asthma, rhinosinusitis, hypertension, and thyroid disease [32]. Other investigations focused on predictors of antihistamine resistance reported that antihistamine-resistant CSU was associated with increased levels of complement C5a fraction in the serum, higher disease activity, longer duration of wheals, and higher positivity of the ASST [80]. Despite these findings, the role of ASST and basophil activation test (BAT) positivity seems to be controversial. Whereas some studies demonstrated the association of ASST and BAT with a poor response to antihistamine treatment [10, 81], others proposed that ASST results are not associated with greater resistance to antihistamine treatment [82]. In a different approach, Asero reported that elevated D-dimer plasma levels should also be considered a marker of antihistamine-resistant chronic urticaria [83]. More recently, adipokines have been a subject of interest in CSU. This is due to the fact that most proinflammatory adipokines are overproduced in metabolic syndrome, while anti-inflammatory adipokines (adiponectin and IL-10) are downregulated [84, 85]. Trinh et al. hypothesized and reported an imbalance between adipokines in CSU. They had observed an increase in mean levels of serum lipocalin-2 (LCN2), TNF-alpha, IL-6, and IL-10 as well as a reduction of adiponectin levels in CSU patients compared to controls. While serum IL-6 levels were significantly higher in refractory CSU patients compared to responsive CSU individuals, the LCN2 levels showed a direct relationship with the urticaria activity score (UAS). Therefore, authors suggested that LCN2 could be a potential biomarker for both disease activity and the clinical responses to antihistamine treatment [86]. A relatively recent study has identified clusterin, a molecule with cytoprotective actions against oxidants, as a predictive biomarker of response to AH [87]. Kim et al. described high levels of clusterin in a group of 69 CSU patients with a positive ASST in contrast to the control group characterized by 69 ASST-negative individuals; moreover, high levels of clusterin seem to be predictive of responsive CSU to AH treatment [87], even if further studies are needed to confirm this evidence (Table 2).

### 4.2. Omalizumab

Omalizumab, an anti-IgE monoclonal antibody, is the first biological agent currently licensed for the treatment of CSU refractory to antihistamine therapy. After 12 weeks of omalizumab treatment, approximately 40% of patients with CSU demonstrated a complete response and 50–70% showed a partial response to therapy. Often, in addition to many nonresponders, there may be a delayed response of up to 6 months until therapeutic benefit is observed, and treatment can be costly [88]. Clinically, most patients with CSU who stop omalizumab treatment relapse within a few months after the last application, and retreatment with omalizumab generally results in rapid remission. It is currently unclear which patient features, if any, are linked to relapse or the time to relapse after omalizumab cessation. Being able to predict which patients will experience rapid symptom return after treatment discontinuation would enable healthcare providers to optimize treatment schedules and facilitate a more informed discussion with patients on their long-term outcome expectations. Herein, the most recent advances in predicting the response to omalizumab therapy will be described (Table 2).

#### 4.2.1. Basophil Histamine Release Assay and ASST

Omalizumab initially complexes soluble IgE and then sequesters IgE released from mast cells, thus uncovering membrane Fc*ε*RI, which subsequently decays slowly over several weeks [89]. It has been postulated that a slow response to omalizumab occurs in CSU patients in whom IgG antibodies to unoccupied IgE receptors (Fc*ε*RI) activate mast cell mediator release causing wheal and angioedema formation [90]. Basophil histamine release assay (BHRA) was used to detect serum autoantibodies directed against either cell-bound IgE or unoccupied Fc*ε*RI. Analysis of omalizumab responders showed that most BHRA-positive patients responded only following the second injection, with a median time to response of 29 days whereas BHRA-negative patients had a median time to response of only 2 days [91]. Furthermore, only 1 of the 39 fast responders was BHRA positive while 8 of the 17 slow responders were BHRA positive (*p* < 0.0001) [91]. This hypothesis was also tested using the ASST [91]. Twelve out of the 33 fast responders were ASST positive, whereas 10 out of the 13 slow responders showed a positive ASST result (*p* < 0.012) [91]. Thus, the authors concluded that a slow response could probably be predicted by a positive BHRA and ASST [91]. There are significant correlations between a positive BHRA and ASST and the time to symptom relief with omalizumab. The fact that a positive BHRA is predictive of a slow response to omalizumab suggests that omalizumab works by reducing Fc*ε*RI expression in those patients.

#### 4.2.2. Levels of Basophil Fc*ε*RI

The potential for predicting clinical outcomes during anti-IgE therapy based on basophil allergen response has been investigated in several studies which mainly focused on pulmonary diseases [92]. It has been demonstrated that the modulation of the basophil Fc*ε*RI expression plays a key role in the clinical improvement observed during omalizumab therapy in CSU [89]. Indeed, a significant drop in basophil Fc*ε*RI expression is observed immediately after the first dose and maintained throughout the duration of the treatment [93]. Deza et al. recently demonstrated in 44 patients the existence of a link between baseline levels of basophil Fc*ε*RI expression and the time to response to omalizumab in CSU, with higher levels shown in patients who responded within 4 weeks (fast responders (FR)). Seventy-five percent of patients were classified as FR and had shown a significantly higher baseline median value of basophil Fc*ε*RI expression than slow responders (SR) (median: 13,247 of MFI and range: 6.7–25.2 versus median: 8428 of MFI and range: 5.7–17.3, respectively, *p* = 0.002, Mann-Whitney *U* test) [94]. However, during the anti-IgE therapy, no significant differences were observed at 4 weeks in the reduction of the Fc*ε*RI expression between fast and slow responders. In the same study, a positive correlation was found between levels of total serum IgE and baseline Fc*ε*RI [94].

The exact mechanisms responsible for fast or slow response remain unknown. Gericke et al. [95] hypothesized that SRs could have autoantibodies directed against Fc*ε*RI (or cell-bound IgE) and that they could interfere with the measurement of basophil Fc*ε*RI expression by flow cytometry, leading to lower detected levels of the receptor than of the FRs. This finding suggests that the Fc*ε*RI downregulation may not be a definitive mechanism of action in some patients and therefore that the combination of more than 1 pharmacological mechanism seems necessary to fully explain the pattern of symptom improvement seen with omalizumab therapy in CSU.

#### 4.2.3. CD203c

CD203c (ectonucleotide pyrophosphatase/phosphodiesterase) is an ectoenzyme which is only expressed on basophils, mast cells, and their CD34 [96] progenitor cells in the peripheral blood [97]. It has been described that CD203c might be a good basophil activation marker due to its specificity and sensitivity [98].

Palacios et al. demonstrated by flow cytometry the ability of CSU patients' serum to activate donor basophils as determined by the upregulation of CD203c [99]; in fact, the lack of basophil CD203c-upregulating activity in the serum of patients with chronic urticaria correlates with a good clinical response to omalizumab [100]. CD203c-upregulating activity was present in 18/41 subjects (43.9%). Of the 18 subjects demonstrating CD203c-upregulating activity, only 9 (50%) experienced clinical improvement with omalizumab. In contrast, of the 23/41 patients without CD203c-upregulating activity, 20 (87%) did have a clinical response to omalizumab (*p* = 0.02, Fisher's exact test). No correlation of efficacy was found with age, sex, or the presence of thyroid autoantibodies (not shown). Omalizumab was effective in 71% of CSU patients overall, slightly higher than what has been reported by other studies [101–104]. Although not proven, the basophil CD203c-upregulating activity is thought to reflect the presence of autoantibodies to IgE and/or Fc*ε*RI*α* suggesting that their presence unexpectedly predicted a lower likelihood of clinical response.

#### 4.2.4. Total IgE Levels

Several studies demonstrated the association between total IgE levels and asthma, atopic dermatitis, and hyper-IgE syndrome; however, in the case of CSU, the correlation seems weaker [105]. Although in 2014, Zheng et al. [106] reported that baseline serum IgE was not predictive of omalizumab clinical response, further analysis of the data does suggest reduced efficacy of this therapy amongst those with the lowest IgE concentrations.

Two retrospective analyses by Metz et al. [103] (*n* = 51) and Viswanathan et al. [107] (*n* = 16) did not show significant differences in serum IgE concentrations between omalizumab responders and nonresponders. However, neither of them stratified low versus normal/high IgE levels and then analyzed for differences in clinical response to omalizumab. Straesser found an association between the lack of serum IgE and lower likelihood of omalizumab response in a multicenter retrospective chart review of 137 patients. When patients were subdivided into serum IgE quartiles (1st: 0–15.2 IU/mL, 2nd: 15.3–68.8 IU/mL, 3rd: 68.9–168.0 IU/mL, and 4th: 168.1–4261 IU/mL), their response to omalizumab differed significantly. Patients with a serum IgE in the 1st quartile had a 48.4% response rate to omalizumab compared with 86.1%, 88.2%, and 94.1% response rates for the 2nd, 3rd, and 4th quartiles, respectively (*p* < 0.001) [107]. In this study, two disease processes have been proposed: one, which is driven by autoreactive IgG and is characterized by female predominance, lower serum IgE, a positive BAT, and less responsiveness to omalizumab. The other, which is IgE mediated (perhaps to self-antigens) and characterized by equal sex prevalence, higher serum IgE, and greater responsiveness to omalizumab [108]. More recently, Ertas et al. confirmed that total IgE levels could have an important role in predicting the clinical response to omalizumab in CSU [109]. They stated that nonresponders to omalizumab had significantly lower baseline IgE levels (bIgE) (mean 17.9, 17.0–55.0 IU/mL) than partial and complete responders (mean 82.0, range 46.2–126.5 IU/mL, and *p* < 0.008 and mean 73.7, range 19.45–153.8 IU/mL, and *p* < 0.032, respectively) [109]. Furthermore, they remarked that nonresponders also had lower IgE levels at week 4 (w4IgE) as well as lower ratios of w4IgE/bIgE levels than partial and complete responders (*p* < 0.001) [109]; for those reasons, they concluded that nonresponse to omalizumab was best predicted by patients' w4IgE/bIgE ratios and was significantly better than by bIgE levels (*p* = 0.016) [109]. This study underlined how IgE levels increase in omalizumab responders during the first weeks of treatment, as was to be expected, whereas IgE levels in nonresponders do not. The reasons for this are unclear; however, one explanation might be that the IgE of nonresponder CSU patients is not bound by omalizumab, since IgE binding and complex formation by omalizumab are held to be the reason for the increase in IgE levels in treated patients. Although free IgE levels were not measured, other studies reported that omalizumab treatment resulted in the reduction of free IgE levels in CSU patients by more than 90% within the first days of treatment [89]. Another possible explanation is that the size of IgE/omalizumab complexes in nonresponders may be smaller than that in responders, resulting in faster clearance of IgE and a lack of increase. The same authors reported how serum levels of total IgE also correlate with time to relapse of CSU after stopping omalizumab treatment. The higher the IgE levels, the less time it takes for CSU symptoms to return after omalizumab is discontinued. Specifically, the time to relapse was significantly shorter in patients with CSU with high IgE levels (>100 IU/mL) compared to those with normal IgE levels [109].

#### 4.2.5. UAS7

In contrast to other studies, Ferrer et al. focused on identifying predictive markers of time to relapse rather than clinical response [110]. In particular, they focused on the speed of response to omalizumab treatment as predictors of rapid symptom return after omalizumab discontinuation. Of 746 variables assessed, two were selected by the model as predictors of symptom return: baseline UAS7 (urticaria activity score over 7 days) and early area above the curve (AAC, determined by plotting the UAS7 scores across time points). Results suggested that a high baseline UAS7 and a low UAS7 AAC (slow decrease of symptoms) indicate higher probability of rapid symptom return than low baseline UAS7 and high UAS7 AAC.

#### 4.2.6. D-Dimer

In a study of 32 patients with CSU, no statistical difference in baseline D-dimer levels was observed between patients with a complete response to omalizumab or with no response [111]. However, D-dimer plasma levels showed a dramatic decrease after only the first administration of omalizumab (from 1024 ± 248 [mean ± SE] to 251 ± 30 ng/mL; *p* = 0.003), and in the majority of patients, D-dimer levels fell within the normal range. In contrast, nonresponders did not show any reduction in D-dimer plasma levels after omalizumab administration. CSU patients with associated angioedema had higher D-dimer plasma levels (1563 ± 467 ng/mL) than CSU patients without angioedema (618 ± 96 ng/mL; *p* = 0.018), and their rate of response to omalizumab was also higher (92 vs. 70%, respectively); however, the difference did not reach statistical significance.

#### 4.2.7. IL-31

Interleukin 31 (IL-31), produced primarily by activated TH2 cells, skin-homing CD45R0 CLA+ T cells, and mast cells, plays an important role in the induction of chronic skin inflammation [112]. It has been reported that serum IL-31 levels of patients with CSU, although not as high as those in atopic dermatitis, are significantly higher than those of healthy controls but not correlated with wheals numbers [113]. Altrichter et al. explored the association between serum IL-31 levels and CSU disease activity in 39 patients before and after 6 months of treatment with omalizumab [114]. Initial IL-31 levels in CSU were very variable (median 230 pg/mL, range 0–30,692 pg/mL) confirming a previous report [113]. In 15 patients treated with omalizumab who showed complete remission of symptoms, the median IL-31 level was significantly (*p* = 0.004) reduced by 48%. Although the numbers were too small for statistical analysis, the median IL-31 levels of the six nonresponders to omalizumab were similar before and after the treatment. Despite the IL-31 level reduction found in patients following successful treatment with omalizumab, the authors do not consider IL-31 to be a primary mediator of itch in CSU due to mismatch between severity of clinical features and IL-31 serum levels.

### 4.3. Cyclosporine

Cyclosporine inhibits cell-mediated immunity by downregulating Th1 lymphocyte responses and T cell-dependent antibody formation by B lymphocytes. In addition, it has inhibitory effects on anti-IgE-induced histamine release from human basophils and skin mast cells in vitro [115, 116]. The presence of a positive ASST response does not appear to be a biomarker for response to cyclosporine [117]. Some studies reported that in patients treated with cyclosporine, a positive BHRA does appear to be a prerequisite for a good clinical response [118]. Furthermore, a shorter duration of the disease and a higher initial severity predict a successful response to treatment with cyclosporine [119]. Asero additionally reported that the D-dimer is a good marker of CSU activity in most patients and that it may be useful in monitoring clinical response to cyclosporine in patients with severe disease [120]. Baek et al., besides confirming these data, excluded any correlation with CRP or serum total IgE [121]. These data along with several previous studies which had found anticoagulation therapy to be effective indicate that coagulation/fibrinolysis may play a role, at least in selected patients, in the pathogenesis of CSU [120] (Table 2).

## 5. Conclusion

Even though several biomarkers are currently being studied, a molecule which is able to help and guide clinicians during the diagnostic process as well as in choice of treatment has not yet been identified. The importance of describing and measuring a poorly understood pathological process such as CSU is growing even more with the increasing therapeutical options which are reaching clinical practice. We emphasize how only an international effort could be successful in generating the amount of data needed to uncover the CSU pathogenesis as well as validating useful biomarkers for the diagnosis and prediction of treatment response.

## Figures and Tables

**Table 1:** Biomarkers of diagnosis, prognosis, and severity.

Biomarker		Diagnosis	Prognosis and severity	
			Better	Worse

Clinical	Age	—	Young pts	Older pts
	Gender	—	Male	Female
	Duration of CSU	—	Less than 1 yr	Longer than 1 yr
	Angioedema	—	Absent	Present
	NSAID exacerbation	—	Not associated	Associated

Molecular	Anti-IL-24 IgE	Positive association	—	—
	IL-6	—	—	High levels
	IL-6 sR	—	—	High levels
	sgp130	—	—	High levels
	IL-18	—	—	High levels
	MMP-9	—	—	High levels
	CRP	—	—	High levels
	D-dimer	—	—	High levels
	F1+2	—	—	High levels
	Vitamin D	—	—	Low levels
	MPV	—	—	High values
	Basophil count	—	Normal number	Low number
	CD203c-basophils	—	—	Increased

MMP-9: metalloproteinase-9; CRP: C-reactive protein; F1+2: prothrombin fragment 1 + 2.

**Table 2:** Biomarkers with a potential prediction on drug treatment.

Biomarker	Antihistamines	Omalizumab	Cyclosporine
*Resistance*			

Clinical	Hispanic	None	None
	Atopic asthma		
	Rhinosinusitis		
	Hypertension		
	Thyroid diseases		
	High UAS7		
	Longer duration of wheals		

Molecular	↑ C5a	BHRA+	None
	↑ IL-6	ASST+	
	↑ D-dimer	Low IgE levels	
	ASST+	CD203c upregulation basophils	

*Response*			

Clinical	None	None	Short duration of CSU
			High initial severity

Molecular	↑ LCN2	BHRA−	BHRA +
	↑ Clusterin	ASST−	D-dimer
		Lack of CD203c basophils	
		High IgE levels	
		High Fc*ε*RI	

*Increased relapse*			

Clinical	None	High UAS7 at baseline	None
		Low UAS7 AAC	

Molecular	None	None	None

LCN2: serum lipocalin-2; BHRA: basophil histamine release assay; ASST: autologous serum skin test; AAC: area above the curve.
